# Characterization of the Oxygen Transmission Rate of New-Ancient Natural Materials for Wine Maturation Containers

**DOI:** 10.3390/foods10010140

**Published:** 2021-01-11

**Authors:** Ignacio Nevares, Maria del Alamo-Sanza

**Affiliations:** 1 Department of Agricultural and Forestry Engineering, UVaMOX, Universidad de Valladolid, Unidad Asociada al CSIC, 34004 Palencia, Spain; 2 Department of Analytical Chemistry, UVaMOX, Universidad de Valladolid, Unidad Asociada al CSIC, 34004 Palencia, Spain

**Keywords:** oxygen transmission rate, permeability, solubility, concrete, earthenware, claystone, granite, *Qvevri*

## Abstract

Today, there is a trend in enology promoting a return to the use of old natural materials for the manufacture of storage and maturation wine tanks. One of the most sought-after characteristics of these materials is their permeability to oxygen from the atmosphere to improve wines without this being a harmful process. The reference performance in wine aging is, without doubt, the oak barrel for its ability to oxidize wines in a controlled way, thus improving them. It would be possible to mature wines in containers in which the use of wood is not obligatory, as opposed to aging in oak barrels or foudres. This work presents the results of oxygen permeation analysis under test conditions typical of a tank containing wine, using materials, such as concrete and granite. The oxygen permeability of the materials tested was very diverse, typical of natural materials. The results showed that earthenware presents an excessive permeability, not only to atmospheric oxygen, but also to liquids and needs treatment before being used in liquid containers. Claystone and concrete can be impermeable to liquids, but maintain permeability to atmospheric oxygen—making them candidates for use in permeable tanks for wine maturation. Finally, granite has some very interesting characteristics, though thickness control is required when calculating the desired oxygen transmission rate.

## 1. Introduction

During storage, wines are exposed to relatively low amounts of oxygen, which are, however, sufficient to influence the results of their maturation or storage. In particular, oxygen modulates the development of the different reactions involving components responsible for the color and aroma of the wine, which results in the formation/degradation of compounds with important consequences on its maturation or storage process. Knowledge of the oxygen permeability of wine aging or storage tanks is of great interest in enology, since the processes of refining and color stabilization require the collaboration of oxygen. The search for alternative materials to the wood used in aging barrels or the need to obtain wines more focused on the fruit than on the wood has raised awareness of different types of materials for these containers. The use of synthetic materials for the construction of these tanks, which interact with the wine as they are permeable to atmospheric oxygen, still deters many wineries in the Old World, due to their fear of damaging the perception of consumers who see aging as a traditional natural process, although they accept technical advances if necessary. Compared to synthetic materials, mainly high-density polyethylene (HDPE) or polydimethylsiloxane (PDMS), the so-called natural materials are an alternative [1]. They can be divided into two classes: Those that are produced on the basis of a formulation of different natural components, such as ceramics (*Qvevri,* terracotta or earthenware), claystone and concrete (considered as a natural material), and, on the other hand, a stone whose composition is not modifiable and depends on its origin though it is a composite. There is a significant demand in the wine sector for information about the behavior of these materials with respect to atmospheric oxygen because the data offered by manufacturers is contradictory and not supported by any scientific evidence.

Measuring permeation as a flux of molecules (permeant) through a material, when there is equal static pressure on both sides of the barrier, but the partial pressure of the permeant gas is different, is called permeability measurement (Fick’s first law of diffusion; Equation (1)), and is the process called diffusion, movement of molecules as a result of random molecular motion when the driving force is the concentration gradient.

(1) $$J=-D\cdot \frac{\delta c}{\delta x}$$

The permeation coefficient (permeability) *P* [m^3^(STP)·m/m^2^·s·Pa] is the product of the diffusion coefficient (diffusivity) *D* [m^2^/s], and Henry’s law solubility coefficient (solubility) *S* [m^3^ (STP)/m^3^·Pa] of the gas in the membrane (Equation (2)), which is the ratio between its concentration *c* [m^3^ (STP)/m^3^] in the membrane and its partial pressure *p* [1/Pa] in the gas phase at a constant temperature [2,3,4] (Equation (3)).

(2) P=D·S

(3) $$S=\frac{c}{p}$$

The flow of O_2_ gas *J* transported per unit of time through the surface of a membrane, also known as Oxygen Transmission Rate (OTR) and the commonly used unit of [cm^3^ (STP)/m^2^·day], is proportional to the constant partial pressure gradient Δ*p* [Pa] between both sides of the membrane (*p_atm_* and *p_0_*) of thickness *L* [m] (Equation (4)).

(4) $$OTR=D\cdot S\cdot \frac{\Delta p}{L}=P\cdot \frac{p_{atm}-p_{0}}{L}$$

Oxygen permeation can then be measured using three methods: Total pressure, isostatic and quasi-isostatic methods [3]. The first method includes the so-called manometrics and volumetrics, in which a pressure difference is generated on both sides of the membrane to be measured and the linear pressure increase is measured when permeation occurs using pressure or volume variation sensors [5,6,7,8]. The isostatic methods establish a difference between the partial pressure of oxygen on both sides of the material to be characterized, while the total pressure on both sides of the material remains similar. The variation in the partial pressure can be measured using oxygen sensors in our case [9,10,11]. Quasi-isostatic methods measure the dynamic accumulation of atmospheric oxygen with optoluminescent sensors [12,13,14], and have proved affordable and equally accurate, since no significant differences between the optical and the carrier gas method have been reported [3]. The coefficient of permeability needs to be measured to have a permanent gas transfer, which is achieved when equal amounts of O_2_ enter and leave the barrier material. In the first stage of the permeability process, the solubility of atmospheric O_2_ in the barrier material is dominant. As the concentration gradient increases on both sides of the material, the second stage of O_2_ diffusion becomes the main contributor to O_2_ permeation [15].

The Time lag method describes how to determine the time needed to obtain steady-state permeability of a gas—taking into account the contribution of diffusion and sorption to overall permeability. One way to speed up the measurement process is to expose both sides of the material to be measured to a vacuum: Once it has been ensured that the gas under study does not exist inside the material, in our case O_2_, the side exposed to the O_2_ of the atmospheric air is subjected to atmospheric pressure, while the other side of the material is balanced with an inert gas at a pressure similar to the atmospheric one. Immediately the O_2_ molecules on the air side begin to solubilize in the material and diffuse towards the free O_2_ side where the increase in the variation of the O_2_ partial pressure is measured. This means that steady-state conditions are not really reached with the dynamic accumulation of O_2_ at a constant volume, since the Δ*p*O_2_ decreases as O_2_ filters through the barrier material. However, since the Δ*p*O_2_ is typically orders of magnitude greater than the partial pressure of the accumulated O_2_, using the term “steady-state conditions” can be justified in this context [16].

**Figure 1:**
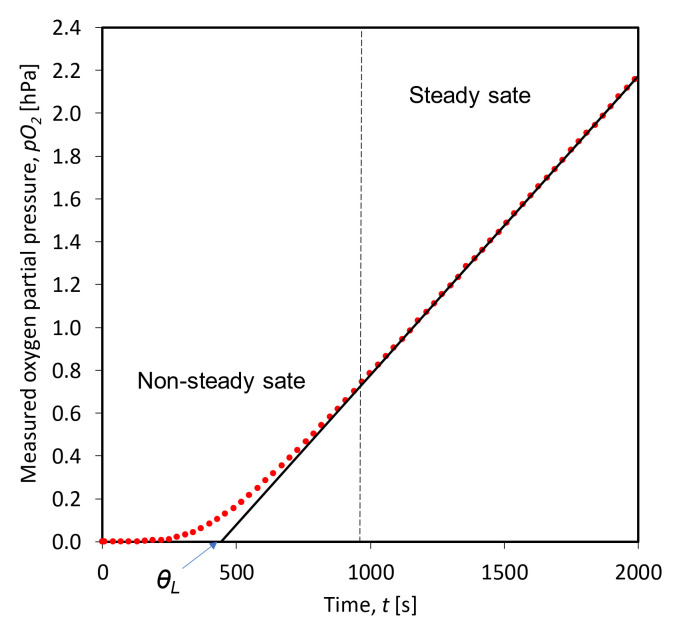
A plot of oxygen partial increment versus time in the oxygen-free chamber.

The outflow time lag, *θ_L_*, is obtained by extrapolating the asymptote of the linear steady-state portion of the pressure profile to the time axis (Figure 1) [17].

In turn, the diffusion coefficient is inversely proportional to the time lag (Equation (5)),

(5) $$D=\frac{L^{2}}{6\theta_{L}}$$

OTR in the steady-state region is calculated from the slope of the increment of the *pO_2_* over time, the volume of the measurement chamber *V* and the surface of the measured membrane *A* considering atmospheric pressure *p_atm_* [18] (Equation (6)),

(6) $$OTR=\frac{\Delta pO_{2}}{\Delta t}\cdot \frac{V}{A\cdot p_{atm}}$$

The permeation coefficient (Equation (7)) is obtained from Equation (4), which also allows the S to be determined thanks to Equation (2).

(7) $$P=OTR\cdot \frac{L}{p_{atm}-p_{0}}$$

**Figure 2:**
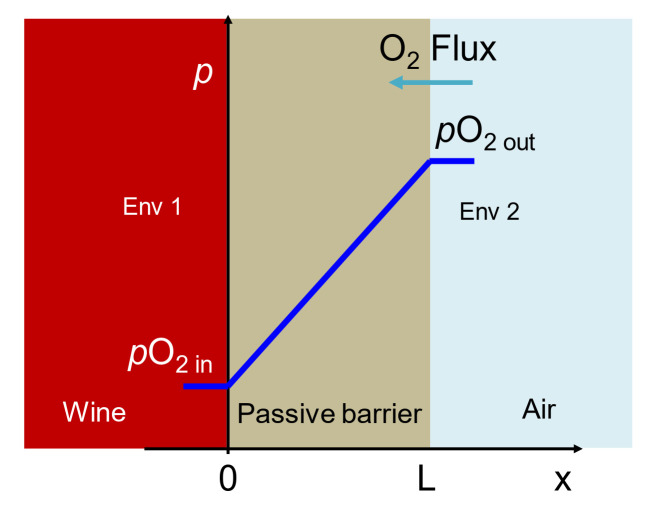
Diffusion scenario in a wine vessel.

Measuring the oxygen permeability or permeation rate of materials different from polymer membranes is approached in many different ways depending on the field. Thus, for concrete oxygen permeability analysis, the common tests are based on the South African Oxygen Permeability Index (OPI) test, in which the time-dependent pressure decay in the permeameter (from an initial value of 100 kPa) is converted to a linear relationship by plotting the logarithm of the ratio of pressure heads versus time [19]. The obtained results coincided with those obtained by other test methods, such as the Cembureau method [20] and the Torrent Permeability Test [21,22]. The study monitors the transfer of that gas for a thickness and a surface over time. In the case of rock, the use of granite in wine storage and maturation tanks has been tested [23,24]. Permeability tests performed on granite also include the scenario of pressure differences on both sides of the tested material thickness [25]. The tests carried out in the analysis of ceramics as a material for the construction of food containers [26,27,28] did use the quasi-isostatic method based on the difference in concentration, something quite common in the analysis of membrane permeability for the food industry. When the permeation rate of oxygen through wood has been determined for use in barrel making [29,30,31,32,33,34], it has been observed to change over time, as wood is porous, so when in contact with wine, its humidity increases, thus lowering the permeation rate over the contact time [35]. Therefore, most of the published data on oxygen permeation of these materials by the aforementioned standards do not correspond to the situation in a container filled with wine, where the main driving force for oxygen inlet is the difference in concentrations on both sides of the wall (Figure 2).

When the material is affected by the infiltration of a liquid modifying its moisture content or by the presence of free water in the porosity, its behavior is substantially modified as in the case of wood [31,32,36]. This situation led to the proposal of a new measurement strategy for the characterization of this type of materials commonly used in the construction of wine maturation and/or storage containers, which are easily infiltrated with the liquid they are in contact with, as is the case with earthenware and concrete. The method proposed is included in patent WO2012107625A1 [23].

The main aim of this study was to test oxygen permeation in materials, such as earthenware or clay, stoneware or claystone, concrete or granite when used for building vessels or tanks for wine maturation. The results of successive tests carried out on different materials over the course of a year indicated that all materials are permeable to atmospheric oxygen and, when used in the right conditions, allow the manufacture of containers suitable for wine maturation.

## 2. Materials and Methods

### 2.1. Materials

#### 2.1.1. Earthenware

**Figure 3:**
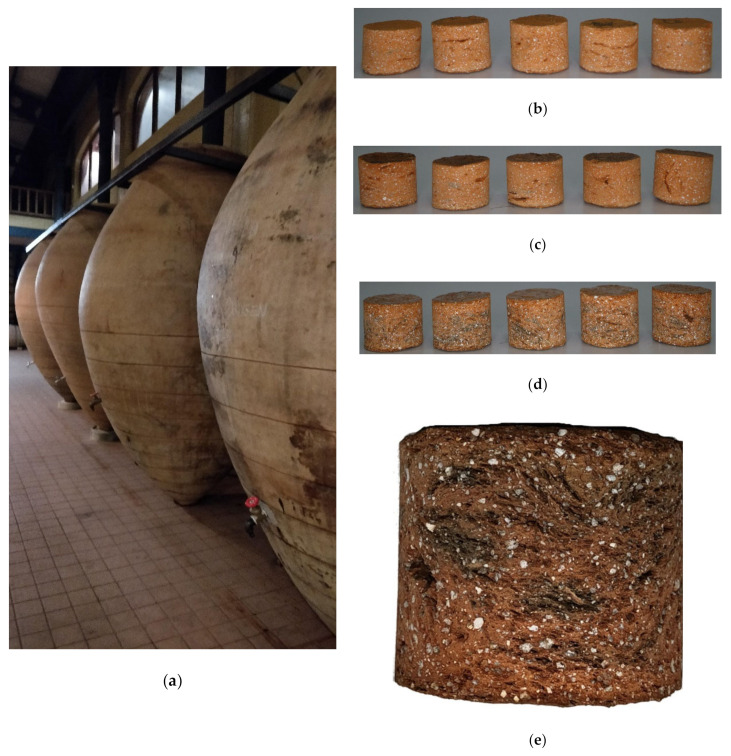
(**a**) Vessels in Prado Rey winery (Burgos, Spain) and (**b**) uncoated raw test specimens; (**c**) specimens coated with 85% colophony + 15% beeswax; (**d**) specimens coated with 85% beeswax + 15% almond oil and (**e**) detail of the composition of the vessel wall.

Clay or earthenware is made of red or white clay fired at a low temperature, typically 1000–1080 °C. As it has not been fired to the point of vitrification, the ceramic is porous and must be subjected to a waterproofing process to contain liquids [37]. This work compiles the results of two types of earthenware. On the one hand, the oxygen permeation behavior of vessels used for fermentation, storage, and aging of wines in Spain, currently being reused in different wineries, was studied. These vessels were traditionally very porous, and many of them were permeable to wine, which required that their inside be covered with beeswax to avoid wine filtration. Three pieces taken from a vessel were received in our laboratory, one of which was studied as a reference, and the other two pieces had two different treatments applied to their inner side, a) 85% beeswax + 15% almond oil (hereinafter, beeswax) and b) 85% colophony +15 % beeswax (hereinafter, colophony). Colophony or rosin is the fresh resin solid fraction obtained from pines after heating to vaporize the volatile liquid terpene components. The treatment was applied to the inner side of the hot pot parts using a brush and heat treatment to ensure the product infiltrated the ceramic. Five samples of each piece were selected for analysis (Figure 3): Five controls, five beeswaxes, and five colophonies, (from now on, EC, Ebee, ECOL samples). They were analyzed when dry and then placed in contact with model wine (pure water and ethanol 12.5% *v*/*v*, pH = 3.5) for seven days on the face that would be located inside the vessel, and these wetted samples were analyzed again.

**Figure 4:**
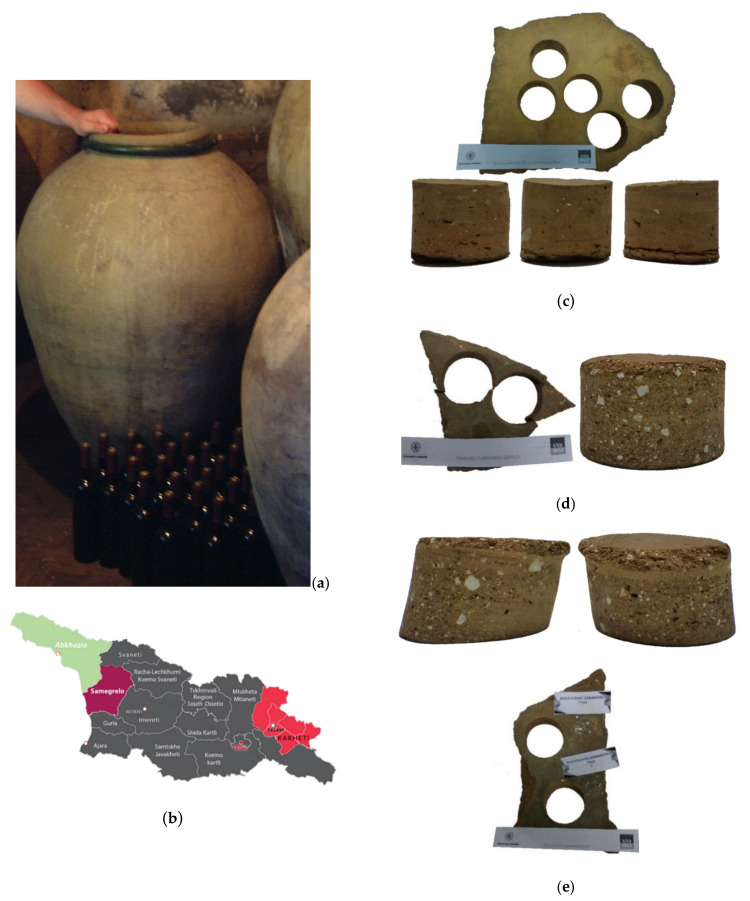
(**a**) Actual *Qvevri* vessels; (**b**) regions of origin of the ceramics tested, Georgia. Vessel pieces and specimens extracted for testing (**c**) Vardisubani near Telavi (Kaheti); (**d**) Abhkazia (Makatubani), and (**e**) Samegrelo.

In addition to these vessels, in a different trial pieces of Georgian vessels (*Qvevri*) from different locations in Georgia were tested: Vardisubani near Telavi (Kaheti); Samegrelo and Abhkazia (Makatubani) (Figure 4; from now on VNT (Vardisubani near Telavi (Kaheti)), SAME (Samegrelo) and ABK (Abhkazia (Makatubani)) samples, respectively).

The ceramic pieces were tested dry and wet. To test *Qvevri* ceramics with coating, the pieces then underwent the classic treatment of waxing with pure beeswax. A protocol was established for their preparation to make the process similar to that followed in the “Kahetian” vinification technique [38]. Both the *Qvevri* pieces and the pure beeswax in a solid state were preheated in an oven at 125 °C until the wax melted. At that moment, the wax was applied to each piece, leaving them for approximately 24 h in the oven at 125 °C, thus keeping the pure beeswax melted on the pieces and facilitating their penetration. The excess wax was then removed, and the pieces were left long enough to cool and to let the wax that had penetrated each piece to solidify.

#### 2.1.2. Claystone

Claystone is composed of ball clay and fire clay, as well as feldspar and silica. Ball clays or plastic clays are fine-grained, highly plastic kaolinitic sedimentary clays, used in the ceramic industry to provide strength and malleability to a ceramic body prior to firing [39]. Fire clay is a normal mudrock, but with a higher alumina (Aluminium(III) oxide, Al_2_O_3_) content. It is fired at high temperatures, typically 1148–1316 °C, and is inherently non-porous, harder, stronger, and more durable than earthenware [37]. White, gray, or brown clay is vitrified during firing; thus, the surface will be practically impermeable to liquids (water absorption ≤ 1%) [40].

**Figure 5:**
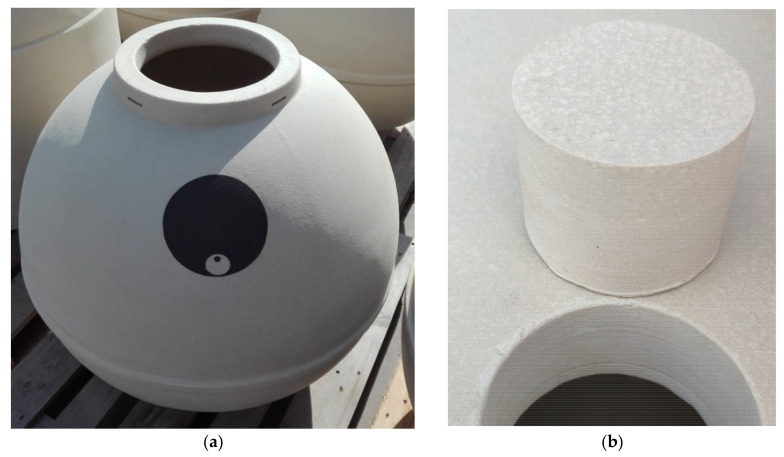

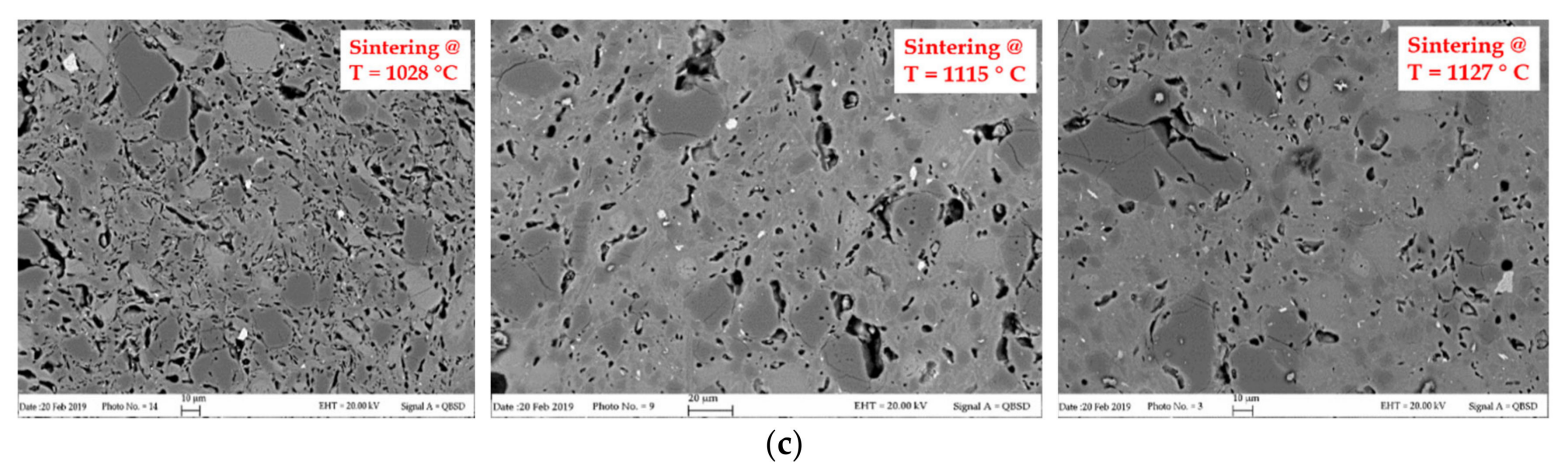
(**a**) 250-L claystone vessel and (**b**) extracted piece for its analysis where the ceramic structure of the material can be observed; (**c**) SEM analyses of different pieces at three sintering temperatures—1028 °C, 1115 °C, and 1127 °C, respectively (images provided by *Clayver*, Savona, Italy).

As the material is waterproof, no special provisions are needed to prevent the liquid from evaporating, such as wax, glaze, or resin coating. The intrinsically microporous structure of the ceramic material allows the exchange of gases with the outside, but, according to the manufacturer, only in limited quantities and over a long time (Figure 5). Claystone may be tailored with controlled heating treatments to offer unique gas and moisture permeation properties [37]. For this work, claystone samples of the necessary dimensions and of three different permeabilities, sintered at different temperatures (Clay 1, Clay 2, and Clay 3 samples) were obtained from a recognized manufacturer of tanks for enology (*Clayver* S.r.l., Savona, Italy).

#### 2.1.3. Concrete

The concrete used in the construction of tanks for winemaking has different compositions, typical of each manufacturer, who build with molds and micro-vibration of the concrete and finally apply a finish inside and outside. The advantages of the tanks built of this material are their natural insulating nature giving them great thermal inertia and their porosity, which can be a disadvantage if not contemplated, although it allows working with micro-oxygenation and without wood. This article records the results of two different analyses of concrete blocks from two well-known manufacturers of tanks in the sector and built utilizing the usual formulation and procedure.

**Figure 6:**
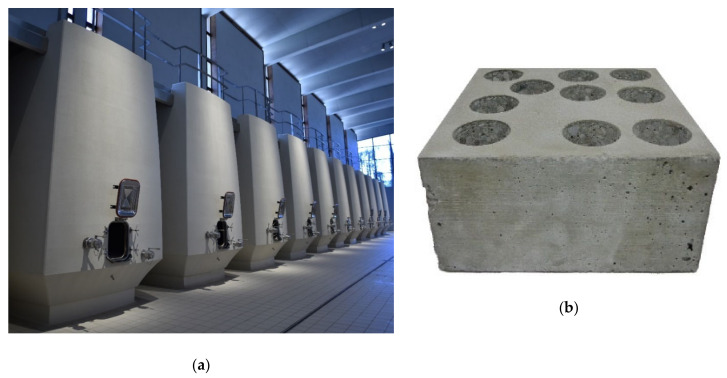

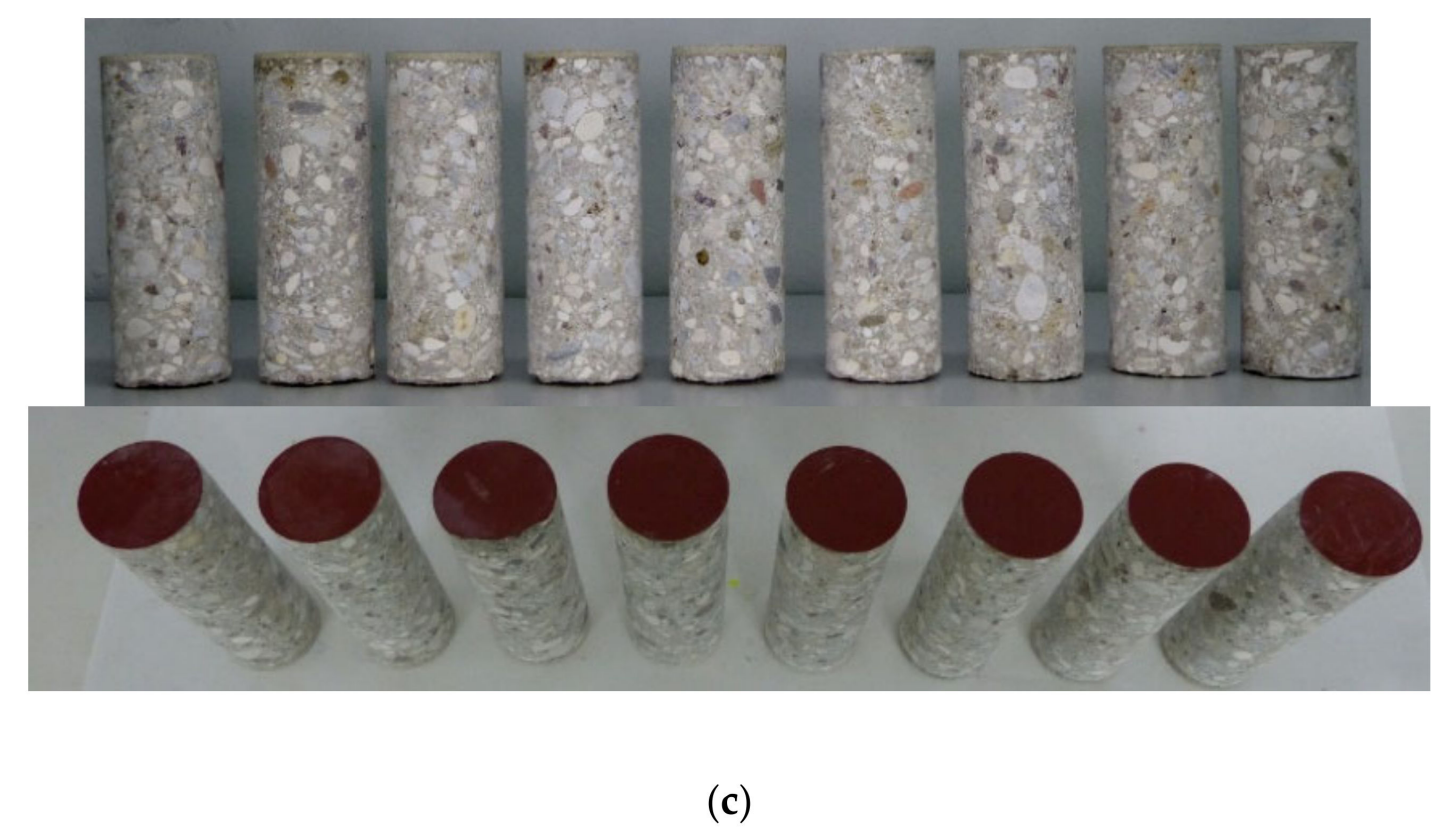
View of (**a**) concrete tanks manufactured with the analyzed concrete (Bodegas Ramón Bilbao, SPAIN), (**b**) view of a concrete block, and (**c**) samples selected for analysis and other similar pieces, but treated with epoxy on the inside.

The first tested concrete comes from the manufacturer of the concrete tanks of Bodegas Ramón Bilbao (Haro, Spain) (Figure 6), while the second one was provided by DVTec (C-DVTec; Saint-Laurent-des-Arbres, France). The thickness of the blocks under study is similar to that used in the tanks. The concrete blocks supplied by Bodegas Ramón Bilbao extracts eight samples (C-RB) of the usual dimensions (see Section 2.2). These samples were analyzed raw and after being tartarized according to the treatment recommended by the manufacturer in the dry fluid mode (see Section 2.3). These tartarized parts were then analyzed under the usual conditions of use (wet fluid mode; see Section 2.4). In addition, an epoxy coated block was received from the manufacturer that analyzes the role that epoxy may have in the OTR of the concrete.

**Figure 7:**
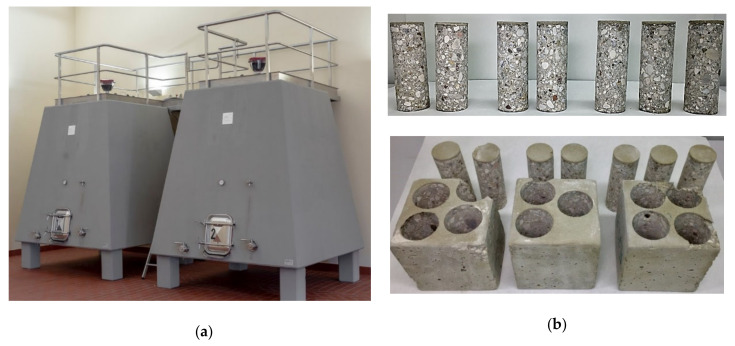
View of (**a**) concrete tanks (DVTec, France) and (**b**) pieces extracted for testing next to the concrete blocks supplied.

The concrete blocks supplied by DVTec extracts seven samples (Figure 7) of the usual dimensions (see Section 2.2). These samples, as the C-RB ones, were analyzed raw and after being tartarized according to the treatment recommended by the manufacturer in the dry fluid mode (see Section 2.3). These tartarized parts were then analyzed under the usual conditions of use (wet fluid mode; see Section 2.4).

#### 2.1.4. Granite

**Figure 8:**
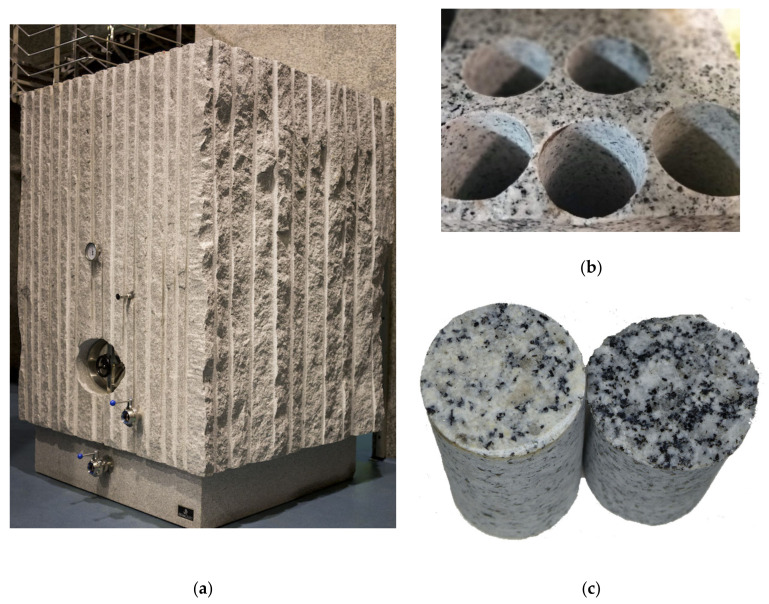
(**a**) View of a granite tank in Mar de Frades winery (Meis, Pontevedra, Spain); (**b**) perforated granite block, and (**c**) granite pieces of the two origins tested where the difference in their composition, especially in mica of the black biotite type, can be observed.

Granite is an igneous plutonic rock formed essentially by feldspar, quartz, mica, and amphibole minerals that occur in different proportions. The fundamental differences between these rocks are based on the size of the crystals, the texture, the conditions of their formation with temperature as the main cause, and which are characteristic of each deposit and its position. In this work, granite rock blocks were obtained from two Spanish deposits in the Iberian Mountain Range, from the Central Iberian zone and from the Galicia zone, seven of clear granite (G-clear) and eight of dark samples (G-dark), the color depending on mica content of the black biotite type (Figure 8). The samples of each granite block (see Section 2.2) were analyzed in time lag mode (see Section 2.3).

### 2.2. Specimen Manufacture and Preparation

To test all the materials, samples were taken using a Hilti DD120 drill (Hilti Inc., Schaan, Liechtenstein) and a 42 mm diameter diamond crown drill. The thickness of the samples was the characteristic of the containers built with each of the materials tested. The sides of the samples were covered with a thin layer of epoxy and a sheet of aluminum to avoid lateral infiltrations and ensure a flow of oxygen in the direction to be tested. Each sample was glued in a stainless-steel cylinder with the necessary connections on both sides of the sample for connection to the test equipment in its time lag configuration or in the fluid model one.

### 2.3. Time Lag Test Setup and Procedure

**Figure 9:**
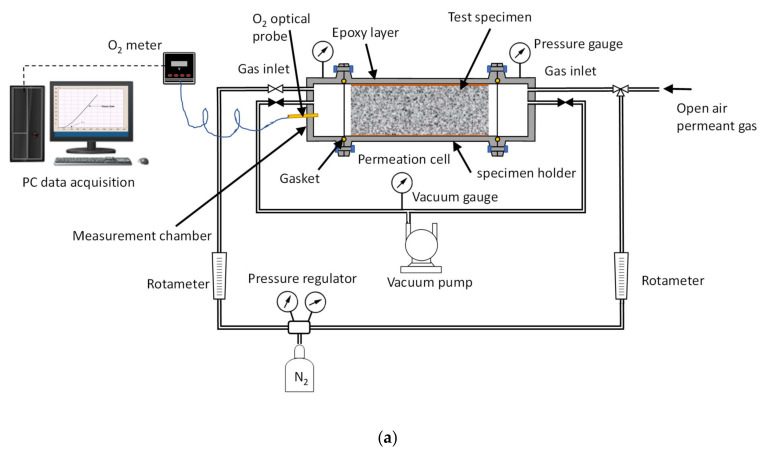

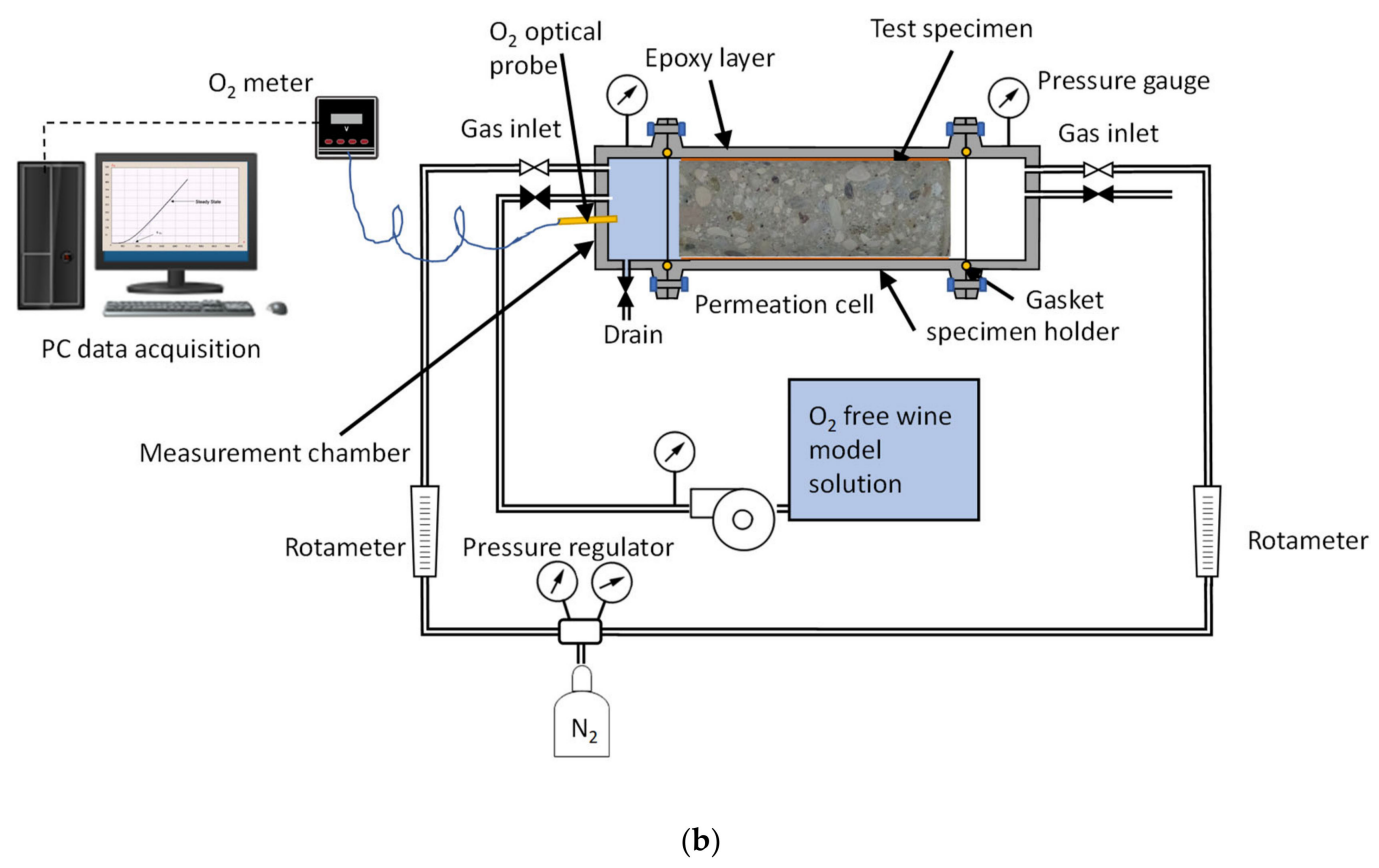
Setup for the specimen tests, (**a**) time lag mode and (**b**) fluid contact mode with gas (dry mode), with the wet material (wet mode) or measuring in model wine instead of in gas (liquid contact mode).

In this test mode, the variation in the oxygen partial pressure in the permeation cell measurement chamber was recorded at constant volume and constant total pressure (Figure 9a). Unlike the other tests, it diffuses the coefficient, and the oxygen solubility coefficient in the material studied to be recorded besides measuring the infiltration rate of the concrete piece and the material (OTR and permeability coefficient). The partial pressure of oxygen pO_2_ was measured using an optoluminescent Fibox 4 Trace meter and a probe fitted with a PSt6 sensor (Presens GmbH, Regensburg, Germany) capable of measuring pO_2_ both in gas and in liquid (Limit of detection 0.002% oxygen in gas and 1 ppb of dissolved O_2_) connected to a PC and controlled by the Presens Measurement Studio 2 software, version 3.0.3 (Presens GmbH, Regensburg, Germany). Prior to the start of the test, successive vacuum phases were performed using an RZ-6 pump controlled by a CVC-3000 controller (Vacuubrand GmbH, Wertheim, Germany), which connected both chambers located on both sides of the sample to be tested. The levels reached during the vacuum phases were 10^−3^ mbar measured by a sensor based on thermal conductivity (Pirani) VSP-3000 (Vacuubrand GmbH, Wertheim, Germany). The first vacuum phase lasted 24 h, followed by the pressure balancing of both chambers with an inert gas, in our case N_2_ (Carburos Metálicos/Airproducts, Marratxí, Spain) at atmospheric pressure. The successive vacuum cycles had a shorter duration, 1 h with N_2_ balancing, until we observed that the materials did not contain air inside by measuring the increase in partial O_2_ pressure for 1 h. Once the vacuum cycles had been carried out and balanced with the inert gas to empty the atmospheric O_2_ from inside the void space of the porosity of the material, the test was started after exposing the opposite side of the measurement chamber of the material to atmospheric air. The whole test was performed in a room at a controlled temperature and relative humidity (RH )(16 °C and 75% RH) with the typical values of a barrel-aging room. All the tests on each sample were repeated three times.

### 2.4. Permeation Test in Fluid, Dry Mode, Wet Mode, and Liquid Contact Mode

Some materials, such as concrete and earthenware, have high porosity and are permeable to liquids, and therefore, need to be treated before they can be used with wine. Their internal surfaces are customarily treated with some layers of different compounds that waterproof against liquids, and it is not known whether the oxygen permeation rate is affected to any extent. For these cases, the measurement installation was modified by replacing the vacuum system with an oxygen-free gas sweep (Figure 9b) with the possibility of performing tests in several ways. The first was in dry mode, in which the variation in the partial pressure of oxygen in the measurement chamber was studied over time. This serve to characterize the material, although it does not reflect the behavior of the material if it is porous and gets wet in contact with the liquid in the container. Thus, tests were carried out with the wet material, conditioned for seven days in contact with model wine, and subsequently measured under the conditions described above, monitoring the variation in the partial pressure of oxygen in the measurement chamber over time. Although this was quite close to reality and allowed a comparison to be made by measuring both situations of the material (dry vs. wet) with the same procedure, it did not fully reflect the real situation of use of a vessel made of that material [41]. To consider this situation, we included the capacity to measure the permeation rate in oxygen-free model wine (H_2_O-EtOH 12.5% *v/v* and pH = 3.5 with tartaric acid), which we will call liquid contact mode. First, both chambers were swept with N_2_ until reaching stable values close to zero (pO_2_ ≤ 1 hPa), and then the measurement chamber was filled with oxygen-free model wine. As in the previous modes, the evolution of the partial pressure of oxygen in the model wine was monitored over time. For the de-gassing model wine, low oxygen levels, a membrane contactor, Liqui-Cel^®^ 4×13 Extra-flow module were used (3M, Maplewood, MN, USA) with fully detailed operation mode in Reference [42].

## 3. Results

### 3.1. Clay or Earthenware

#### 3.1.1. Earthenware from Spanish Amphorae

Spanish pottery samples from the mid-20th century presented physical properties similar to the ceramic pieces from the Georgian *Qvevri* vessels (thickness: 29.28 ± 1.55 mm; weight: 56.94 ± 2.4 g and density: 1.89 ± 0.02 g/cm^3^). These samples were analyzed in the fluid contact mode arrangement (Figure 9b) by measuring first with nitrogen (dry mode) and then with the material moistened with model wine (wet mode). The results are shown in Table 1.

#### 3.1.2. *Qvevri* from Georgia

*Qvevri* Georgian ceramics presented different characteristics according to their origin, as they were at least 50 years old. Many of them presented problems during sample extraction, and only a few of each type could be collected (Table 2). The ceramics from Vardisubani near Telavi (Kaheti) (VNT) were observed to have a higher density, and a greater thickness and probably greater volume, and the samples from the Samegrelo (SAME) and Abhkazia (Makatubani) (ABK) areas presented very similar values.

We tried to characterize the samples of vessels from the three Georgian regions using tests with the set-up in its time lag mode (Figure 9a), but this was impossible, due to their excessive porosity. For this reason, it was decided to carry out the study in the fluid contact mode set-up (Figure 9b), measuring first with nitrogen (dry mode) and then with the material moistened with model wine (wet mode) (Table 3).

### 3.2. Claystone or Stoneware

Several samples of sintered claystone were sent to our laboratory to analyze permeation to oxygen, as well as several properties involved in the diffusion process. The main characteristics are shown in Table 4.

Each of the clay types included two replicate samples, and all of them were tested in dry mode simultaneously to avoid any interference in the testing conditions, mainly barometric pressure, although it was automatically compensated. The main results of the measurements are shown in Table 5.

After the gas analysis, the six samples of three different types were tested in the Liquid contact mode set-up, which records their behavior in conditions similar to those of real use. Table 6 shows the results obtained.

### 3.3. Concrete

#### 3.3.1. Concrete samples C-RB

In the case of C-RB concrete, numerous samples were taken from various blocks. Table 7 shows its main properties.

**Figure 10:**
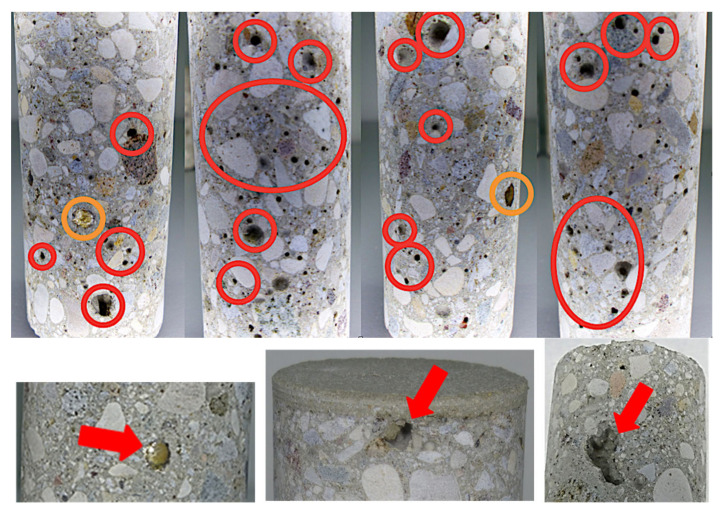
Detail of the variation in the composition of the material and highlights of the pieces of metal (orange) and the air holes (red) that will undoubtedly affect the permeability performance of the concrete block.

It is interesting to note that many of the samples extracted showed small cavities typical of the trapping of air bubbles during the construction process, which can partially explain the differences that may appear between samples from the same block. Figure 10 shows these bubbles, usually small, but sometimes larger. In principle, their influence should be limited as long as they are far from the interior face of the tank in contact with the wine. The results of the measurements in time lag mode, both for the raw samples and once they had received the tartaric acid coating treatment on their inner face, are shown in Table 8.

The tartaric samples were then analyzed in the liquid contact mode, which reproduces the conditions closest to those of actual use by measuring the evolution of dissolved oxygen within a model wine in contact with the tartaric acid conditioned face. After one week in contact with the liquid, the pieces were tested. The results are shown in Table 9.

In addition, the eight pieces with epoxy coating were tested in the time lag mode, and in all cases, the oxygen input was negligible and considered impermeable to atmospheric oxygen for enological purposes.

#### 3.3.2. Concrete Samples C-DVTec

In the case of the concrete blocks provided by DVTec, seven samples were taken for subsequent analysis, the main characteristics of which are shown in Table 10. In this case, the voids were barely visible, unlike the samples taken from the other manufacturer’s concrete blocks.

The first analysis was made in the fluid contact mode set-up (Figure 9b), measuring with nitrogen first (dry mode) and with model wine afterward (wet mode). The measurement in wet mode was made after seven days in contact with model wine. After these samples were dried to their initial weight, they were tartarized, as described in Section 2, and tested again in both dry and wet modes. The values obtained are shown in Table 11.

Once the tests in the dry and wet fluid mode were completed and after a period of the tartaric acid conditioned samples in contact with the model wine, they were tested in the Liquid contact mode, which reproduced the conditions closest to the real conditions of use when the evolution of dissolved oxygen is measured within a model wine in contact with the tartaric acid conditioned face. The results can be seen in Table 12.

### 3.4. Granite

Different samples of each granite block about 250 mm thick from the two areas studied were analyzed and extracted using the same procedure as for the other materials analyzed. To carry out a study in the designed device, the thickness needed to be adjusted to the usual one in the deposits of this type of material, thus allowing comparison. All the pieces were adjusted to a thickness of 100 mm. The details are shown in Table 13.

The granite pieces were analyzed according to the time lag method, as water absorption was found to be practically negligible after wetting tests. This characteristic made them candidates for this type of test, which, compared to the other modes, also allowed the diffusion coefficient and the solubility coefficient to be characterized. The results of this analysis are shown in Table 14.

Once the tests in the fluid dry mode were completed, they were tested in the Liquid contact mode, which reproduces the conditions closest to real conditions of use when measuring the evolution of dissolved oxygen within a model wine. The results can be seen in Table 15.

## 4. Discussion

### 4.1. Clay or Earthenware

The results of the first dry test with the untreated earthenware samples from Spanish amphorae showed very high OTR values (Table 1), even higher than those of the Georgian ceramic samples (Table 2 and Table 3), possibly due to the former’s lower thickness. On the other hand, earthenware samples from Spanish amphorae with beeswax and colophony treatment showed much lower OTR results, demonstrating the barrier effect produced by the coating even with the dry material. When the samples were tested in wet mode, the OTR decreased considerably. Although the OTR data measured in wet mode seems to indicate that the lowest values were obtained with the EC pieces, it is necessary to emphasize that this occurs because many of the pieces tested were completely wet in such a way that the entire thickness of the ceramic was with all its porosity flooded. The oxygen diffusion was completely slowed down, and the model wine in those pieces was wetting its exterior face. This indicates that without the appropriate coating treatment, the container would lose part of the liquid, even if only by evaporation, causing a cooling effect sought in other types of porous containers. The results of the permeability coefficient of the earthenware pieces studied (Table 1 and Table 3) are of the same order as those obtained in Korean *Onggi* ceramic tests (6.85·10^−14^ to 7.59·10^−12^ m^3^∙m/m^2^∙s∙Pa), although in their case the walls were much less thick (4 mm) using different clay formulations and glazing treatments [26].

Varying permeability coefficients were observed in *Qvevri* samples from different regions, the highest being those of VNT origin ceramics. It should be remembered that the permeability coefficient is a characteristic of the raw material, regardless of the thickness of the ceramic. When carrying out permeation measurements on samples of a given thickness, the flow of atmospheric oxygen through the vessel wall still differed, even though the thickness of the vessel wall was greater. When the vessel walls were dry, the ceramic VNT raw material was characterized by a P slightly more than double that of SAME and ABK (Table 2). When analyzing the samples from the vessels, since the VNT samples are almost 40% thicker than the SAME and ABK vessel samples (Table 2), the OTR differences remained.

When the samples were moistened with model wine, only on the side that would be inside the vessel, a strong drop in OTR was observed (Table 3). The entry of O_2_ decreased as would be expected, since it is a porous material. As in oak barrel staves [31,32], the resistance to oxygen transmission increased in the concrete [43,44,45] or in the Korean *Onggi* ceramic when wet [27,28]. The decrease is also affected by the density of the material, so the OTR_wet_ in VNT pieces was only 0.71% of the OTR_dry_, while the other two ceramics reflected a lower percentage decrease than the VNT (OTR_wet-SAME_ = 0.93% OTR_dry-SAME_ and OTR_wet-ABK_ = 1.71% OTR_dry-ABK_) (Table 2).

If the vats were used in their original state, the enormous model wine absorption would make them completely inappropriate for storing wine, since in some pieces, the liquid would drip through to the outside of the vat. The ceramics were dried in an oven to a constant weight, and then pure beeswax was applied following the procedure described in Section 2.

**Figure 11:**
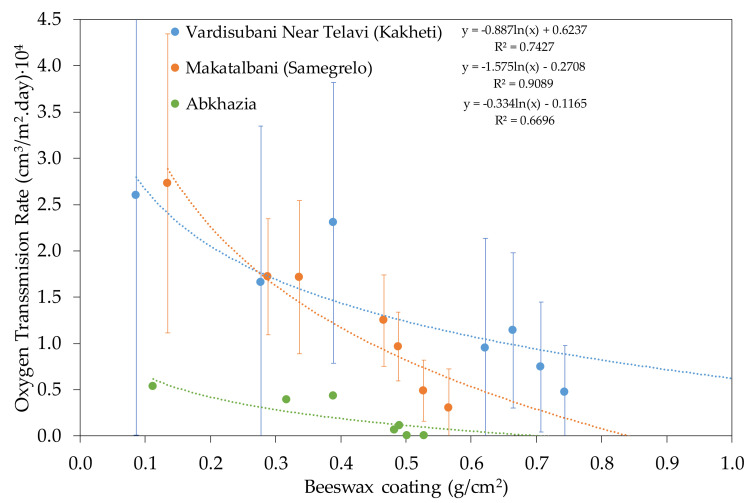
Evolution of OTR in the pieces of *Qvevri* from different regions as a function of beeswax coating.

As commented in previous sections, the inner face of Georgian ceramic vessels was treated with wax and the amount of wax affected the oxygen permeability of the material. Figure 11 shows the decrease in OTR of the three types of ceramics from the different Georgian regions analyzed as a function of the amount of wax applied. This decrease varied among the three types of pieces, depending on the physical properties of the ceramic. Thus, the VNT ceramic, although the densest, initially gained less weight of wax on average during the treatments. However, after several days of treatment, it collected more wax, although it had the lowest porosity, due to its higher density. In spite of this, the only ABK sample presented the greatest drop in OTR, and thus, being the densest material, received less wax and consequently presented the least variation in OTR in successive treatments with wax. The successive wax treatments on the SAME samples allowed for a decrease in their OTR. The VNT ceramic samples with greater thickness and lower density could probably take on more wax, and thus, decrease their OTR.

### 4.2. Claystone

**Figure 12:**
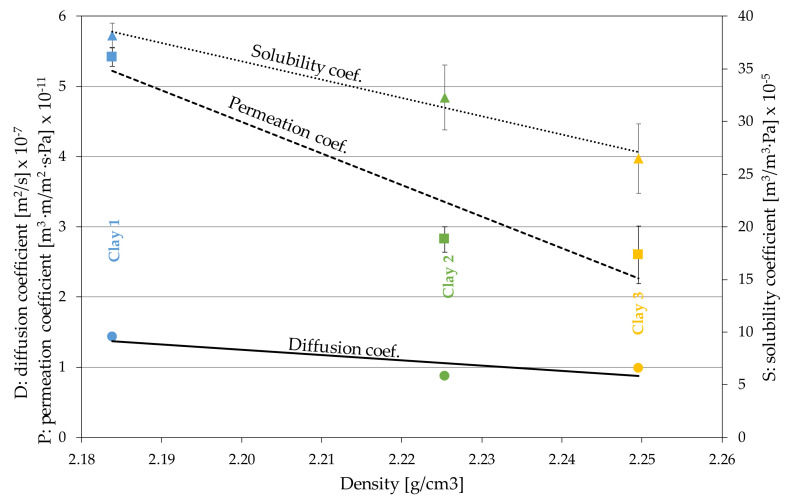
Variation of permeation properties as a function of density in claystone pieces.

The production of Claystone with different sintering temperatures allows pieces of different fired densities to be obtained (Table 4), which determine open porosity and provide the ceramic with a different permeability to water vapor [46]. In our analysis of O_2_ permeation, it can be seen that, as in the study of physical characterization of ceramics [46], the solubility of oxygen depended on the fired density, and therefore, the porosity is logical. In the same way, the permeation coefficient decreased with the fired density, reducing the diffusion and, as a result, claystone of different OTR properties can be obtained without changing the thickness of the piece or structurally modifying the material or its behavior towards atmospheric O_2_ (Figure 12).

Given the low liquid absorption reported by the manufacturer [46], the material was tested under the measurement conditions closest to the real situation, in contact with model wine (Liquid contact mode Figure 9b). It was observed that the behavior of the pieces with different densities was very similar to that obtained in the tests in dry fluid mode (Table 5 and Table 6), although the absolute values obtained were much lower. This shows that measuring under conditions similar to those of real use is necessary to quantify the OTR of the material. The low water absorption means a constant oxygenation rate during the aging time can be assumed, which differentiates this material from the dynamic operation of oak wood in barrels [33].

*Clayver* tanks have been tested with OTR rates of 12.96 mg/L.year [1]; it would, therefore, be possible to manufacture vessels with half the current rates.

### 4.3. Concrete

The solubility of oxygen in concrete quantifies the volume of oxygen that a volume of concrete can take up. As shown in Table 8, the solubility coefficient of raw concrete C-RB decreased by 12% when the concrete was tartarized. The origin of this was attributable to the decrease in surface porosity of the internal face of the concrete, which resulted in a lower oxygen absorption by the concrete. The diffusion coefficient increased by 25% with a corresponding slight increase (10%) in the permeability coefficient. It is interesting to highlight the studies that analyzed the permeability of concrete to gases [22,43], following test methods that use a difference in an oxygen pressure of 1 bar on both sides of the concrete sample [20,47,48,49,50], a situation that is governed by Darcy’s law. This is hardly comparable to the oxygen diffusion that occurs during the storage of wines in this type of tanks that is governed by Fick’s Law. On measuring the characterization of C-RB dry concrete as a material, it can be stated that, far from affecting it negatively, tartarization does not diminish the capacity of oxygen permeation. On the contrary, the C-DVTec concrete pieces tested in dry-mode showed permeability coefficient values several orders of magnitude lower than the C-RB concrete pieces (Table 8 and Table 11). This shows that concrete composition, both the proportion and the type of cement and aggregates, determine its gas permeability. That has been tested and shown in previous studies [51,52], despite the fact that different methodologies were used. The properties of C-DVTec concrete pieces were greatly affected by the tartaric acid treatment, which could be explained by the fact that it is a concrete of lower density and more porosity, thus would presumably have allowed tartaric acid to penetrate more into the treated face.

**Figure 13:**
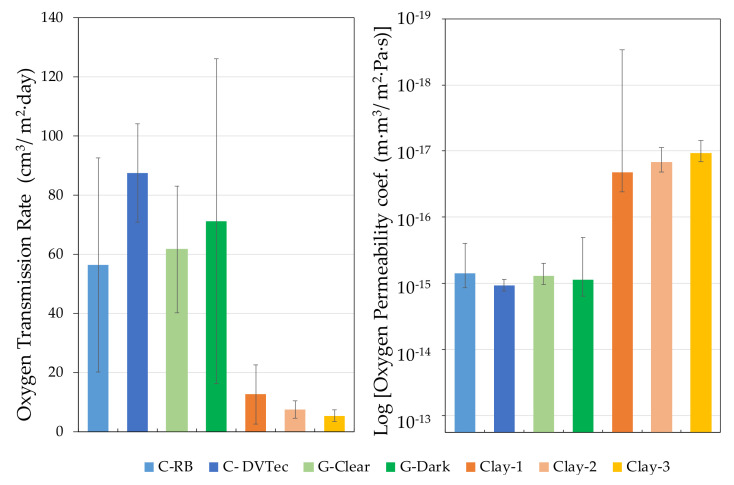
Variation of permeation properties in liquid contact mode (Tartaric acid conditioned for concrete).

When C-DVTec parts were wetted, their coefficient of permeability clearly decreased whether they were tested in wet mode (Table 11) or in liquid contact mode (Table 12). When untreated concrete parts were wetted for seven days in contact with model wine and then measured again, there was a huge decrease (99.98%), as one would expect from a very porous material. This was somewhat mitigated when treated with Tartaric acid (88.44% decrease), although the reduction was still dramatic. Although this type of test allows us to reflect the behavior of materials that become wet and increase their water or wine content in our case, it does not really represent real use situations. For this reason, and thanks to the liquid contact mode of the test device (Figure 9b) and the capacity of the equipment to measure the partial pressure of oxygen in both gas and liquid, measurements were made under the usual conditions of use (treated with tartaric acid and in contact with a model wine) on both types of concrete. The permeability coefficient under these conditions decreased greatly in both types. The C-DVTec pieces had a permeability coefficient similar to that of the C-RB pieces (Figure 13). Thus, the heterogeneity of the composition of the pieces tested (Figure 6c and Figure 7b) showed that the percentage of aggregates and gravel marked both the permeability and the OTR, and a more or less homogeneous mixture of the components of the concrete would explain the variation in the results. Therefore, the characterization of small diameter samples, as was the case, required a sufficient number of samples to guarantee the correct characterization of the material.

### 4.4. Granite

The granite samples had the same dimensions as the concrete ones, although they presented a higher density than those or the claystone ones, and this probably affected their permeability, since the porosity was lower. Both types of granite, although different, presented density values very close to each other, as shown in Table 13. If we compare it with the rest of the materials, in this case, the natural material, granite, presented values of physical properties with higher consistency than the formulated materials. As for the properties of the permeation test in the time lag method, the permeability coefficient (2.79–28.2·10^−11^ m^3^∙m/m^2^∙s∙Pa) was of the same order of magnitude as that of claystone (2.60–5.42·10^−11^ m^3^∙m/m^2^∙s∙Pa) and much lower than that of concrete C-RB (6.58–7.24·10^−8^ m^3^∙m/m^2^∙s∙Pa), also tested in this mode. Granite, as an igneous plutonic rock, cooled slowly from high temperatures (between 1215–1260 °C) under high pressure and only claystone, sintered at temperatures between 1085–1127 °C and cooled slowly, is comparable since earthenware has done so at lower temperatures and concrete is not a fired material. The low gas permeability of granite makes it an option for housing spent nuclear fuels in deep geological repositories [53]. In studies carried out in this area it has been postulated that it can also reduce and buffer the ingress of oxygen-rich waters [54], so, although it has not been calculated experimentally, the low OTR values measured in both types of granite could be explained, especially in the results in liquid contact mode (Table 15). The differences found for both types of granite are not statistically significant, although other studies established that when a higher amount of biotite was available, the penetration of oxygen was slightly lower [55].

### 4.5. Comparison of Materials for the Construction of Wine Tanks

The characterization of these materials extrapolates the behavior of different tanks for enology manufactured with these materials and predicts their potential performance in real conditions of use in the winery. For this purpose, the data obtained in the Liquid contact mode tests have been used, on both types of concrete (C-RB and C-DVTec) tartarized on their inner side, on the three types of claystone analyzed, and on the two types of granite tested. The measurements obtained in the model wine were transformed, considering the solubility of oxygen in water, and the relationship between the external surface of the deposits and the volume of wine that would fit in different geometric shapes was estimated. For this purpose, different manufacturers were consulted, and the ratio of surface area (m^2^)/volume of wine (L) was determined for each shape and size. The results obtained from the OTR of these materials were applied for thicknesses of 10 cm, although each manufacturer may vary both the thickness and the formulation of the concrete. In the case of granite, being the only really natural product analyzed, each block of rock will have different characteristics depending on its origin and the situation of the piece in the deposit. Only in the case of claystone was the thickness provided by the manufacturer used.

**Figure 14:**
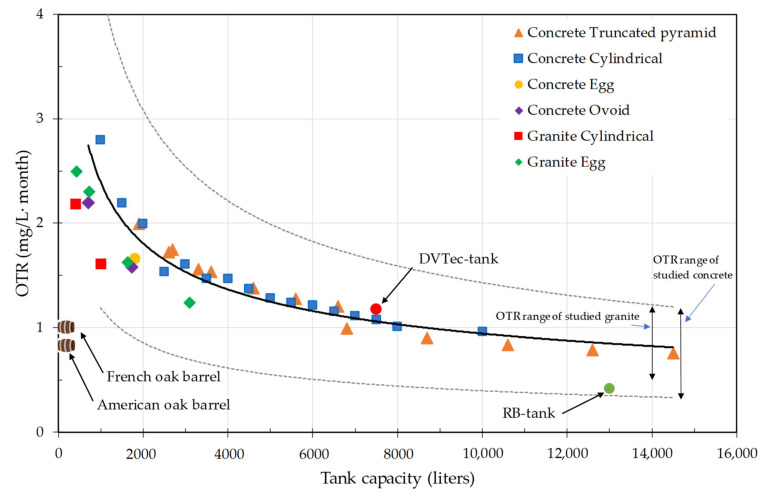
Variation of monthly average OTR (mg/L∙month) in tartaric acid conditioned concrete tanks for the average measured OTR in liquid contact mode (equivalent to real working conditions), and for granite tanks based on dimensional specifications of commercial tanks and different shapes. The possible contribution of the different elements (valves, lids, etc.) of a tank to the OTR was not considered. Dotted lines refer to the mean ± SD.

Figure 14 compares the different OTRs, obtained in mg/L·month, for containers of different volumes made with each of the materials measured under conditions of use (concrete and granite), and all of them offer conditions that make them very interesting for the maturation of finished wines or for their aging, with or without wood. They all presented OTR values higher than those of the barrel. It is important to note that the OTR of the barrel is the average of the annual OTR, which has been reported as dynamic [32,33]. In addition, if the wood is used as an alternative for maturing wines in these tanks, the oxygen supplied by the addition of wood, if any, would have to be added. For example, if a contribution of oxygen similar to that of a barrel is being sought, a concrete with a low OTR should be used (in the lower range of the concretes studied) regardless of the volume of the tank, as can be seen in Figure 14. Therefore, the size factor does not seem to be limiting in the case of concrete tanks. The type of concrete will clearly mark the difference in the evolution of the wine, since the difference factor between both types of concrete is 3, when comparing both types in tartarized tanks and without the application of epoxies or external treatments to avoid dirtying or for decoration common in this type of tank. Clearly, the claims of some manufacturers of concrete tanks that the OTR of their tanks is negligible must be disregarded, unless they have been treated with specific coatings to prevent this.

As far as granite containers are concerned, the shape factor is not limiting, though the thickness and the vein of rock with which it is built are. It seems an interesting material for the construction of wine tanks and micro-oxygenation that some manufacturers defend: Although not quantified, it is a probability. If an approximation is made of the values obtained in the tests in Liquid contact mode of the average value of both types of granite, and the volume ratio is applied to the granite tanks marketed in a cylindrical shape, or to the granite barrels of different volumes, it can be seen that they are within the general trend of all these permeable tanks (Figure 14). The OTR values obtained for both types of granite (Table 15), although they are of the same order as those obtained for both types of tartarized concrete (Table 9 and Table 12), their average value is below the average OTR of the concrete. For this reason, they have lower OTR values for similar volumes (Figure 14). In general, for small volume containers, all the materials studied to provide a higher OTR than the average annual value of the barrels. This does not mean that in the first moments, the values offered by the barrels may be higher than those of the tanks of natural permeable materials, although later, the barrels may dramatically lower their OTR [34]. As for the containers built with claystone, it is the most technified material because not only its composition, but also the sintering process are controllable, which produces containers with a controlled OTR. Finally, containers built with earthenware have the disadvantage of requiring, for the most part, treatments to prevent the leakage of the liquid they contain, which limits their OTR before these containers can be used for maturing and aging wines.

In the near future, a new generation of ceramic containers will be available in which the firing temperature, along with the formulation of the clay will be the key to obtaining controlled and known OTR vessels. The development of concrete formulations in search of OTR control seems to be an interesting field for manufacturers. To avoid MOX, there is always the internal epoxy coating, which has been shown to minimize OTR making it negligible in C-RB pieces. The very nature of granite will mean that the construction of tanks made of this material will produce containers with different OTRs, which are difficult for the manufacturer to manipulate except for the choice of block or the thickness of the material used. More research is needed to explore the possible role that some of the granite components could have in reducing and buffering the OTR, and that could somehow modulate the oxidative aging process of the wine by influencing the redox state of the rock-wine combination.

## 5. Conclusions

The tests were carried out on samples with the real thicknesses of the tanks and containers manufactured using the different materials, which allowed the OTR values in a situation similar to real use to be recorded.

All the materials studied have a certain permeability to oxygen and because of the values obtained, they are suitable for use in the construction of containers for enology without spoiling the wine by excess oxygenation or reducing the wine, due to a defect—as happens in the stainless-steel tanks widely used in all wineries around the world.

The real oxygen rate provided to the wine by tanks made of different materials must be quantified under conditions that reproduce the real scenario, in our case, the liquid contact mode.

Claystone presents ideal values for the oxidative maturation of wines, and the OTR is perfectly controllable using the sintering temperature, although it seems that it can be a limiting factor in the size of the containers. The same applies to earthenware containers, which require an internal treatment to prevent leakage of the liquid and to slow down the entry of atmospheric oxygen. The volume of the containers also seems to be limited by the nature of the material.

Concrete has been postulated as an ideal material for the manufacture of large tanks with the capacity to mature wine, but the formulation of the concrete and the execution in the construction of the container are key aspects to graduating the intake of atmospheric oxygen. The application of exterior or interior coatings to the concrete will undoubtedly alter its OTR, even to the point of being considered negligible, as in the case of the application of epoxy.

Granite appears to be an interesting material—both for its capacity to micro-oxygenate the wine it contains and for the possible influence it may have on redox phenomena depending on the nature of its composition. The size of the container is limited by the density of the material and by the size of the rock needed to work the whole container as a single piece if so desired.

## Figures and Tables

**Table 1:** Main permeation characteristics of tested Spanish earthenware samples with different coating treatments.

Mode	OTR·10^−4^ (cm^3^/m^2^·day)			Permeability Coef.·10^13^ (m^3^·m/m^2^·s·Pa)		
	EC (*n* = 5)	Ebee (*n* = 5)	Ecol (*n* = 5)	EC (*n* = 5)	Ebee (*n* = 5)	Ecol (*n* = 5)
Dry	517.35 ± 117.83 (22.8%)	109.85 ± 84.50 (76.9%)	215.23 ± 116.99 (54.4%)	175407 ± 36257 (20.7%)	39093 ± 30273 (77.4%)	81169 ± 46658 (57.5%)
Wet (t = 7 days)	0.12 ± 0.03 (25.0%)	2.78 ± 2.60 (93.5%)	14.65 ± 25.26 (172.4%)	0.23 ± 0.08 (34.8%)	0.50 ± 0.35 (70.0%)	22.42 ± 42.83 (191.0%)
Liquid absorption (g)	4.92 ± 0.29 (5.9%)	2.58 ± 2.33 (90.3%)	3.86 ± 2.06 (53.4%)			
Moisture content (%)	8.96 ± 0.45 (5.0%)	4.51 ± 4.06 (90.0%)	6.51 ± 3.39 (52.1%)			

Mean ± SD; coefficient of variation in brackets. OTR: Oxygen Transmission Rate; EC: earthenware control; Ebee: earthenware coated with beeswax and Ecol: earthenware coated with colophony.

**Table 2:** Main physical characteristics of tested *Qvevri* samples from different regions in Georgia.

Material	Diameter (mm)	Thickness (mm)	Weight (g)	Density (g/cm^3^)
VNT (*n* = 4)	30.92 ± 1.33 (4.3%)	33.16 ± 0.46 (1.4%)	70.81 ± 0.99 (1.4%)	1.80 ± 0.01 (0.5%)
SAME (*n* = 3)	30.43 ± 0.49 (1.6%)	24.43 ± 0.49 (2%)	49.70 ± 0.16 (0.3%)	1.70 ± 0.02 (1.2%)
ABK (*n* = 1)	31.07	24.27	49.97	1.73

Mean ± SD; coefficient of variation in brackets. VNT (Vardisubani near Telavi (Kaheti)), SAME (Samegrelo) and ABK (Abhkazia (Ma-katubani)).

**Table 3:** Main permeation characteristics of tested *Qvevri* samples from different regions in Georgia.

Mode	OTR·10^−4^ (cm^3^/m^2^·Day)			Permeability Coef.·10^14^ (m^3^·m/m^2^·s·Pa)		
	VNT (*n* = 4)	SAME (*n* = 3)	ABK (*n* = 1)	VNT (*n* = 4)	SAME (*n* = 3)	ABK (*n* = 1)
Dry	30.97 ± 5.37 (17.3%)	11.75 ± 10.30 (87.7%)	12.79	122.97 ± 21.03 (17.1%)	48.50 ± 8.08 (16.7%)	37.19
Wet (t = 7 days)	0.22 ± 0.10 (45.4%)	0.11 ± 0.10 (90.9%)	0.22	0.87 ± 0.39 (44.8%)	0.47 ± 0.05 (10.6%)	0.64
Liquid absorption (g)	5.39 ± 0.31 (1.8%)	4.21 ± 0.70 (16.6%)	2.81			
Moisture content (%)	7.62 ± 0.50 (6.6%)	8.48 ± 1.42 (16.7%)	5.62			

Mean ± SD; coefficient of variation in brackets.

**Table 4:** Main physical characteristics of tested clay samples (*Clayver* data).

Material	Furnace T (°C)	Diameter (mm)	Thickness (mm)	Weight (g)	Density (g/cm^3^)	Absorbed Water (1 h)
Clay 1 (*n* = 2)	1100	39.05	16.00	41.83	2.18	2.79%
Clay 2 (*n* = 2)	1110	39.50	15.98	43.55	2.22	1.93%
Clay 3 (*n* = 2)	1120	39.50	15.99	44.06	2.25	1.23%

**Table 5:** Main permeation results of tested claystone samples.

Material	Diffusion Coefficient (m^2^/s) D 10^7^	OTR (cm^3^/m^2^·Day)	Permeability Coef. (m^3^·m/m^2^·s·Pa) P∙10^11^	Solubility Coef. (m^3^/m^3^·Pa) S∙10^5^
Clay 1 (*n* = 2)	1.43 ± 0.0155 (1.1%)	612.64 ± 15.38 (2.5%)	5.42 ± 0.136 (2.5%)	38.17 ± 1.18 (3.1%)
Clay 2 (*n* = 2)	0.877 ± 0.0288 (3.3%)	319.01 ± 20.51 (6.4%)	2.82 ± 0.181 (6.4%)	32.26 ± 3.08 (9.5%)
Clay 3 (*n* = 2)	0.983 ± 0.0298 (3.0%)	293.63 ± 46.14 (15.7%)	2.60 ± 0.408 (15.7%)	26.47 ± 3.32 (12.5%)

Mean ± SD; coefficient of variation in brackets.

**Table 6:** Main permeation results of tested claystone samples in liquid contact mode.

Material	OTR (cm^3^/m^2^∙Day)	Permeability Coef. (m^3^∙m/m^2^∙s∙Pa) P 10^17^
Clay 1 (*n* = 2)	12.57 ± 10.02 (79.2%)	2.09 ± 2.06 (98.6%)
Clay 2 (*n* = 2)	7.41 ± 2.91 (39.3%)	1.47 ± 0.58 (39.3%)
Clay 3 (*n* = 2)	5.38 ± 1.93 (35.9%)	1.07 ± 0.39 (35.9%)

Mean ± SD; coefficient of variation in brackets.

**Table 7:** Main physical results of tested C-RB samples.

Material	Diameter (mm)	Thickness (mm)	Weight (g)	Density (g/cm^3^)
Average (*n* = 8)	36.23 ± 0.05 (0.1%)	103.35 ± 0.83 (0.8%)	244.35 ± 2.10 (0.9%)	2.29 ± 0.009 (0.4%)

Mean ± SD; coefficient of variation in brackets.

**Table 8:** Main permeation results of tested samples extracted from concrete blocks supplied by Bodegas Ramón Bilbao (C-RB) (*n* = 8) in time lag mode.

Material	Diffusion Coef. (m^2^/s) D∙10^7^	OTR∙10^−9^ (cm^3^/m^2^∙Day)	Permeability Coef. (m^3^∙m/m^2^∙s∙Pa) P∙10^8^	Solubility Coef. (m^3^/m^3^∙Pa) S∙10^7^
No treatment	3.69 ± 0.42 (11.40%)	1.08 ± 0.03 (2.55%)	6.58 ± 0.17 (2.55%)	18.77 ± 2.17 (11.54%)
Tartaric acid conditioned	4.60 ± 0.53 (11.60%)	1.19 ± 0.07 (5.59%)	7.24 ± 0.38 (5.31%)	16.51 ± 1.65 (9.98%)

Mean ± SD; coefficient of variation in brackets.

**Table 9:** Main permeation results of tested C-RB pieces in liquid contact mode.

Material	OTR (cm^3^/m^2^∙day)	Permeability Coef. (m^3^∙m/m^2^∙s∙Pa) P∙10^16^
Tartaric acid conditioned (*n* = 8)	56.43 ± 36.24 (64%)	7.00 ± 4.50 (64.3%)

Mean ± SD; coefficient of variation in brackets. C-RB: The concrete blocks supplied by Bodegas Ramón Bilbao extracts eight samples.

**Table 10:** Main physical results of tested pieces extracted from concrete blocks provided by DVTec (C-DVTec).

Material	Weight (g)	Diameter (mm)	Thickness (mm)	Density (g/cm^3^)
Average (*n* = 7)	233.00 ± 1.37 (0.59%)	36.13 ± 0.03 (0.10%)	102.86 ± 0.57 (0.55%)	2.21 ± 0.01 (0.44%)

Mean ± SD; coefficient of variation in brackets.

**Table 11:** Main permeation results of tested C-DVTec pieces in Fluid mode, dry and wet, (*n* = 7).

Mode	No Treatment	Tartaric Acid	No Treatment	Tartaric Acid
Dry	**OTR (cm^3^/m^2^·day)·10^−3^**		**Permeability coef. (m^3^·m/m^2^·s·Pa)·10^15^**	
	407.57 ± 131 (32.18%)	0.18 ± 0.12 (69.59%)	2480 ± 799 (32.20%)	1.09 ± 0.76 (69.99%)
Wet	**OTR (cm^3^/m^2^·day)·10^8^**		**Permeability coef. (m^3^·m/m^2^·s·Pa)·10^16^**	
	5.16 ± 1.74 (33.67%)	1.22 ± 0.66 (54.32%)	5.42 ± 1.82 (33.68%)	1.26 ± 0.68 (53.70%)

Mean ± SD; coefficient of variation in brackets.

**Table 12:** Main permeation results of tested C-DVTec pieces in liquid contact mode.

Material	OTR (cm^3^/m^2^∙Day)	Permeability Coef. (m^3^∙m/m^2^∙s∙Pa) P∙10^15^
Tartaric acid conditioned (*n* = 7)	87.54 ± 16.65 (19%)	1.08 ± 0.21 (22.3%)

Mean ± SD; coefficient of variation in brackets.

**Table 13:** Main physical results of tested granite pieces.

Material	Diameter (mm)	Thickness (mm)	Weight (g)	Density (g/cm^3^)
G-Clear (*n* = 7)	36.18 ± 0.04 (0.10%)	99.16 ± 2.60 (2.63%)	264.92 ± 7.26 (2.74%)	2.60 ± 0.01 (0.42%)
G-Dark (*n* = 8)	36.10 ± 0.07 (0.18%)	99.97 ± 0.94 (0.95%)	269.94 ± 3.40 (1.26%)	2.64 ± 0.01 (0.30%)

Mean ± SD; coefficient of variation in brackets. G-clear: clear granite and G-dark: dark granite.

**Table 14:** Main permeation results of tested granite pieces in time lag mode.

Material	Diffusion Coef. (m^2^/s) D 10^8^	OTR∙10^−6^ (cm^3^/m^2^∙Day)	Permeability Coef. (m^3^∙m/m^2^∙s∙Pa) P∙10^11^	Solubility Coef. (m^3^/m^3^∙Pa) S∙10^9^
G-Clear (*n* = 7)	8.33 ± 2.05 (26.6%)	1.83 ± 0.21 (11.4%)	2.79 ± 0.74 (26.6%)	3.34 ± 0.23 (7.0%)
G-Dark (*n* = 8)	13.8 ± 17.5 (126.7%)	1.03 ± 0.062 (6.01%)	28.2 ± 4.73 (16.7%)	3.74 ± 1.17 (31.2%)

Mean ± SD; coefficient of variation in brackets.

**Table 15:** Main permeation results of tested granite pieces in liquid contact mode.

Material	OTR (cm^3^/m^2^∙Day)	Permeability Coef. (m^3^∙m/m^2^∙s∙Pa) P 10^16^
G-Clear (*n* = 7)	61.63 ± 21.36 (34.7%)	7.67 ± 2.72 (35.5%)
G-Dark (*n* = 8)	71.21 ± 54.89 (77.1%)	8.80 ± 6.76 (76.8%)

Mean ± SD; coefficient of variation in brackets.

## Data Availability

The data presented in this study are available on request from the corresponding author. The data are not publicly available due to the fact that they belong to the different companies collaborating in the cession of the materials.

## References

[1] Nevares I., Del Alamo-Sanza M. (2018). New materials for the aging of wines and beverages: Evaluation and comparison. Food Packaging and Preservation.

[2] De Angelis M.G., Drioli E., Giorno L. (2014). Solubility coefficient (S). Encyclopedia of Membranes.

[3] Müller K., Scheuerer Z., Florian V., Skutschik T., Sängerlaub S. (2017). Comparison of test methods for oxygen permeability: Optical method versus carrier gas method. Polym. Test..

[4] Larsen H., Kohler A., Magnus E.M. (2000). Ambient oxygen ingress rate method. An alternative method to Ox-Tran for measuring oxygen transmission rate of whole packages. Packag. Technol. Sci..

[5] Annu. B., American Society for Testing and Materials D1434-82 (2021). Standard Test Method for Determining Gas Permeability Characteristics of Plastic Film and Sheeting.

[6] International Standard Organization (ISO) 15105-1 (2007). Plastics—Film and Sheeting—Determination of Gas-Transmission Rate—Part 1: Differential-Pressure Methods.

[7] German Institute for Standardization DIN 53380-2:2006-11 (2006). Testing of Plastics—Determination of Gas Transmission Rate—Part 2: Manometric Method for Testing of Plastic Films.

[8] German Institute for Standardization DIN 53380-1:2000-07 (2000). Testing of Plastics—Determination of Gas Transmission Rate—Part 1: Volumetrical Method for Testing of Plastic Films.

[9] American Society for Testing and Materials D3985-05 (2005). Standard Test Method for Oxygen Gas Transmission Rate Through Plastic Film and Sheeting Using a Coulometric Sensor.

[10] German Institute for Standardization DIN 53380-3: 1998-07 (1998). Testing of Plastics—Determination of Gas Transmission Rate—Part 3: Oxygen-Specific Carrier Gas Method for Testing of Plastic Films and Plastics Mouldings.

[11] International Standard Organization (ISO) 15105-2 (2003). Plastics—Film and Sheeting—Determination of Gas-Transmission Rate—Part 2: Equal-Pressure Method.

[12] German Institute for Standardization DIN 53380-5:2014-12 (2014). Testing of Plastics—Determination of Gas Transmission Rate—Part 5: Optical Method for Plastic Films and Moulded Plastic Parts.

[13] American Society for Testing and Materials F3136-15 (2015). Standard Test Method for Oxygen Gas Transmission Rate through Plastic Film and Sheeting Using a Dynamic Accumulation Method 1.

[14] American Society for Testing and Materials F2714-08(2013) (2008). Standard Test Method for Oxygen Headspace Analysis of Packages Using Fluorescent.

[15] Lomax M. (1980). Permeation of gases and vapours through polymer films and thin sheet—part I. Polym. Test..

[16] Al-Ismaily M., Wijmans J., Kruczek B. (2012). A shortcut method for faster determination of permeability coefficient from time lag experiments. J. Membr. Sci..

[17] Shah M.R., Noble R.D., Clough D.E. (2007). Measurement of sorption and diffusion in nonporous membranes by transient permeation experiments. J. Membr. Sci..

[18] Diéval J.-B., Vidal S., Aagaard O. (2011). Measurement of the Oxygen Transmission Rate of Co-extruded Wine Bottle Closures Using a Luminescence-Based Technique. Packag. Technol. Sci..

[19] Salvoldi B., Beushausen H., Alexander M. (2015). Oxygen permeability of concrete and its relation to carbonation. Constr. Build. Mater..

[20] Kollek J.J. (1989). The determination of the permeability of concrete to oxygen by the Cembureau method—A recommendation. Mater. Struct..

[21] Torrent R., Frenzer G. A method for rapid determination of the coefficient of permeability of the “covercrete”. Proceedings of the International Symposium Non-Destructive Testing in Civil Engineering.

[22] Torrent R.J. (1992). A two-chamber vacuum cell for measuring the coefficient of permeability to air of the concrete cover on site. Mater. Struct..

[23] Philipp C., Schödl H., Sari S., Korntheuer K., Patzl-fischer- E. (2019). Influence of different storage containers on chemical and sensory fingerprint of Pinot blanc and Grüner Veltliner wines. Mitteilungen Klosterneubg.

[24] Schödl H., Schweighofer H., Herzog R. (2017). Betonei, Steinfass, Tonamphore & Co. Vergleich mit herkömmlichen Weinbehälter. Der Winzer.

[25] Skoczylas F., Henry J.P. (1995). A study of the intrinsic permeability of granite to gas. Int. J. Rock Mech. Min. Sci. Geomech. Abstr..

[26] Seo G., Chung S., An D., Lee D. (2005). Permeabilities of Korean earthenware containers and their potential for packaging fresh produce. Food Sci. Biotechnol..

[27] Seo G.H., Yun J.H., Chung S.K., Park W.-P., Lee D.S. (2009). Physical properties of korean earthenware containers affected by soy sauce fermentation use. Food Sci. Biotechnol..

[28] Yun J.H., An D.S., Lee K.-E., Jun B.S., Lee D.S. (2006). Modified atmosphere packaging of fresh produce using microporous earthenware material. Packag. Technol. Sci..

[29] Nevares I., Del Alamo-Sanza M. (2015). Oak stave oxygen permeation: A new tool to make barrels with different wine oxygenation potentials. J. Agric. Food Chem..

[30] Nevares I., Del Alamo-Sanza M., Martínez-Martínez V., Menéndez-Miguélez M., Bulcke J.V.D., Van Acker J. (2019). Influence of Quercus petraea Liebl. wood structure on the permeation of oxygen through wine barrel staves. Holzforschung.

[31] Nevares I., Crespo R., Gonzalez C., Del Alamo-Sanza M. (2014). Imaging of oxygen transmission in the oak wood of wine barrels using optical sensors and a colour camera. Aust. J. Grape Wine Res..

[32] Nevares I., Mayr T., Baro J. (2016). Á; Ehgartner, J.; Crespo, R.; Del Alamo-Sanza, M. Ratiometric oxygen imaging to predict oxygen diffusivity in oak wood during red wine barrel aging. Food Bioprocess Technol..

[33] Del Alamo-Sanza M., Cárcel L.M., Nevares I. (2017). Characterization of the Oxygen Transmission Rate of Oak Wood Species Used in Cooperage. J. Agric. Food Chem..

[34] Del Alamo-Sanza M., Nevares I. (2014). Recent Advances in the Evaluation of the Oxygen Transfer Rate in Oak Barrels. J. Agric. Food Chem..

[35] Del Alamo-Sanza M., Nevares I. (2017). Oak wine barrel as an active vessel: A critical review of past and current knowledge. Crit. Rev. Food Sci. Nutr..

[36] Del Alamo-Sanza M., Nevares I., Mayr T., Baro J. (2016). Á; Martínez-Martínez, V.; Ehgartner, J. Analysis of the role of wood anatomy on oxygen diffusivity in barrel staves using luminescent imaging. Sens. Actuators B Chem..

[37] Piergiovanni L., Limbo S. (2016). Food packaging materials. Mechanism of Functional Expression of F1-ATPase.

[38] Barisashvili G. Qvevri making in Georgia, history and present. Making Wine in Qvevri—A Unique Georgian Tradition. Proceedings of the 1st International Qvevri Wine Symposium; Georgian Wine Association.

[39] Manning D.A.C. (1995). Introduction to industrial minerals. Introduction to Industrial Minerals.

[40] Martín-Márquez J., Rincón J.M., Romero M. (2008). Effect of firing temperature on sintering of porcelain stoneware tiles. Ceram. Int..

[41] Del Alamo M., Nevares I. (2012). Device for Measuring the Permeability and Diffusivity of Gases in Porous Materials and Method for Measuring Said Parameters Using the Device. World Intellectual Property Organization Patent.

[42] Prat-García S., Nevares I., Martínez-Martínez V., Del Alamo-Sanza M. (2020). Customized oxygenation barrels as a new strategy for controlled wine aging. Food Res. Int..

[43] Real S., Bogas J.A. (2017). Oxygen permeability of structural lightweight aggregate concrete. Constr. Build. Mater..

[44] Kameche Z., Ghomari F., Choinska M., Khelidj A. (2014). Assessment of liquid water and gas permeabilities of partially saturated ordinary concrete. Constr. Build. Mater..

[45] Villani C., Loser R., West M.J., Di Bella C., Lura P., Weiss W.J. (2014). An inter lab comparison of gas transport testing procedures: Oxygen permeability and oxygen diffusivity. Cem. Concr. Compos..

[46] Peroni V., Botter R., Cabella R., Risso L., Carbone C., Bernardo C., Petti F.M., Innamorati G., Fascio L. (2019). Mineralogical and microstructural characterization of stoneware for wine use. Il tempo del Planeta Terra e il Tempo dell’Uomo: Le Geoscienze tra Pasato e Futuro, Proceedings of the Congresso Nazionale Parma, Parma, Italy, 16–19 September 2019.

[47] AI-Otaibi O.M., Barr B. (2002). Comparative study of three permeability tests. Proceedings of the 27th Conference on Our World In Concrete & Structures.

[48] Buenfeld N.R., Okundi E. (1998). Effect of cement content on transport in concrete. Mag. Concr. Res..

[49] Wong H., Zobel M., Buenfeld N., Zimmerman R. (2009). Influence of the interfacial transition zone and microcracking on the diffusivity, permeability and sorptivity of cement-based materials after drying. Mag. Concr. Res..

[50] Zhu W., Bartos P.J. (2003). Permeation properties of self-compacting concrete. Cem. Concr. Res..

[51] Wong H.S., Zimmerman R.W., Buenfeld N. (2012). Estimating the permeability of cement pastes and mortars using image analysis and effective medium theory. Cem. Concr. Res..

[52] Tittarelli F. (2009). Oxygen diffusion through hydrophobic cement-based materials. Cem. Concr. Res..

[53] Trinchero P., Sidborn M., Puigdomenech I., Svensson U., Ebrahimi H., Molinero J., Gylling B., Bosbach D., Deissmann G. (2019). Transport of oxygen into granitic rocks: Role of physical and mineralogical heterogeneity. J. Contam. Hydrol..

[54] Sidborn M., Neretnieks I. (2007). Long term redox evolution in granitic rocks: Modelling the redox front propagation in the rock matrix. Appl. Geochem..

[55] Malmström M., Banwart S. (1997). Biotite dissolution at 25°C: The pH dependence of dissolution rate and stoichiometry. Geochim. Cosmochim. Acta.

